# Examining the psychometric properties of the Coach-Athlete Relationship Questionnaire (CART-Q) with basketball players in China and Spain

**DOI:** 10.3389/fpsyg.2023.1273606

**Published:** 2023-10-19

**Authors:** Jingfei Wang, Carlos Cordente-Martínez, Jonathan Ospina-Betancurt

**Affiliations:** ^1^Departamento de Deportes, Facultad de Ciencias de la Actividad Física y del Deporte (INEF), Universidad Politécnica de Madrid, Madrid, Spain; ^2^Departamento Didáctica de la Expresión Musical, Plástica y Corporal, Facultad de Educación, Universidad de Valladolid, Valladolid, Spain; ^3^Valoración del Rendimiento Deportivo, Actividad Física y Salud, y Lesiones Deportivas (REDAFLED), Universidad de Valladolid, Soria, Spain

**Keywords:** closeness, commitment, complementarity, validity, reliability, culture

## Abstract

The study examines the complex interactions between coaches and athletes in federative basketball in two different cultural contexts: China and Spain. The paper examines the interpersonal psychological dimensions from a direct viewpoint and a meta-perspective, drawing on the Coach-Athlete Relationship Questionnaire (CART-Q) and guided by the 3Cs model (Closeness, Commitment, and Complementarity). The CART-Q was translated and modified for use in both nations’ federative basketball organizational systems to guarantee cross-cultural applicability. Careful translation techniques were used to achieve semantic homogeneity between the Spanish and Chinese versions of the questionnaire, including talks with knowledgeable linguists. The translated versions improved response comparability and kept the same item numbers as the original 2012 instrument. Out of the 771 distributed questionnaires, 763 legitimate answers were gathered via online surveys conducted using secure platforms (Google Forms for Spain and Wenjuanxing for China). The research included a three-step mediation study using structural equation modeling, which allowed for a thorough investigation of the concurrent validity of the modified CART-Qs. The findings indisputably support the reliability and validity of the CART-Qs translated into Chinese and Spanish. The research shows small but significant cultural disparities in the comprehensive perspective of coach-athlete interactions. These results have extensive ramifications for the sport and highlight how crucial it is to consider cultural differences when coaching and developing athletes.

## 1. Introduction

The quality of the coach-athlete relationship is a vital component of sports coaching since it significantly impacts athletes’ performance, success, and general happiness. In this setting, Yang and Jowett’s (2012) Coach-Athlete Connection Questionnaire (CART-Q) and the 3Cs paradigm (Closeness, Commitment, and Complementarity) have emerged as crucial instruments for assessing and comprehending this connection. Despite their usefulness, there are still uncertainties about the universality and applicability of these models in many cultural contexts. Recent studies, such as one by King (2021), highlight the crucial part that the coach-athlete relationship plays in determining an athlete’s motivation, commitment, and performance outcomes. The 3Cs model provides a strong framework that fully reflects this relationship’s complex nature and includes proximity, commitment, Complementarity, and co-orientation. Based on this paradigm, the CART-Q has been created as a reliable instrument for evaluating the caliber of interactions between coaches and athletes.

Giulianotti (2015) points out that much validation research for these models has mostly focused on British coaches and players. Therefore, looking at how the coach-athlete relationship shows itself in various cultural contexts is crucial. There may be considerable regional differences in coaching methods, athlete communication, and the importance of interpersonal relationships. The paper focuses on two different cultural contexts–China and Spain–each offering unique social and cultural aspects that might significantly impact the dynamics of coach-athlete interaction.

Draw attention to the study results of Lupo et al. (2017) to emphasize the relevance of coach-player interactions and the possibility of various perspectives within this dynamic. Lupo et al. (2017) highlight the complex nature of coach-athlete interaction by demonstrating how athletes might view basic components of their training experiences differently from their instructors. Their research focused on top young female basketball players. The 3Cs, or closeness (emotions), commitment (cognitions), and complementarity (behaviors) are the three separate aspects that make up the CART-Q.

The emotional aspects of the athlete-coach connection are explored in depth by the Closeness dimension, which includes things like shared understanding, support, and trust. By examining this factor, researchers learn more about the strength of the relationship and rapport developed between athletes and their coaches, revealing the degree of emotional closeness within the dyad. The Commitment component, on the other hand, examines the athlete’s commitment and faith in the knowledge and experience of the coach. It explores the cognitive components of the partnership, such as how the athlete views the coach’s skill and dedication to the coaching process. Insights on the coach’s influence on motivation, attitude, and belief in training and performance may be gained by understanding the athlete’s degree of commitment.

The last component, Complementarity, evaluates the behavioral aspects of the athlete-coach connection. It examines how athletes and coaches cooperate and coordinate their efforts to achieve shared objectives. This dimension provides important information on cooperation and understanding between people, two factors that greatly impact how well training tactics work and how well people perform. Researchers have found the CART-Q’s standardized framework to be very helpful in conducting thorough examinations into how the coach-athlete relationship affects performance- and wellbeing-related outcomes. The validity and reliability of its measures have been confirmed via prior research (Yang and Jowett, 2012), solidifying its status as a reliable evaluation tool in studies examining the dynamics of coach-athlete interaction.

This research aims to expand knowledge of the 3Cs paradigm and the application of the CART-Q across various cultural settings, acquiring insights into culturally sensitive coaching techniques. It also intends to assess the internal reliability and factorial validity of the CART-Q in samples of top athletes from China and Spain. To assess the concurrent validity of the CART-Q, the relationship between athletes’ levels of sport pleasure is also examined. Additionally, consideration is given to how, in various cultural circumstances, empathy could help clarify how coaches and players interact. The research also aims to considerably expand understanding of the coach-athlete relationship, its evaluation, and its effects on athlete performance via a thorough cross-cultural investigation. The research will help design coaching tactics attentive to cultural differences to improve athletic performance and general wellbeing among different racial and ethnic groups.

## 2. Methodology

### 2.1. Participants

Total responses of 763 federated players, 384 (50.3%) represent China (55.6% men and 44.4% women), and 379 (50.3%) from Spain (61.4% men and 37.6% women). The participants were between 14 and 35 years, with a mean age of 22.39 (SD = 4.74). This range is justified because the sample size is generally considered the population required to obtain meaningful and statistically significant results with a 95% confidence level and no more than a 5.03% margin of error (Brysbaert, 2019).

### 2.2. Procedure

The research began by obtaining player contact information from various sports organizations and leagues. To ensure privacy and compliance with data protection regulations, personalized invitation letters were sent to potential participants, emphasizing the voluntary nature of participation. Each participant provided informed consent after reviewing a comprehensive consent form detailing the research objectives and confidentiality measures. The questionnaire was then distributed online through secure platforms, using Google Forms for participants in Spain and Wenjuanxing for those in China.

Out of the 771 questionnaires received, 763 valid responses were identified and included in the dataset after careful review to ensure data accuracy. Data collection followed a sequential batch approach, efficiently managing many participants and facilitating organized analysis. Rigorous data analysis was conducted using the Statistical Package for the Social Sciences (SPSS), allowing various statistical tests to explore relationships and patterns within the data.

Secondary data analysis focused on reputable sources published within the last 10 years to complement the primary findings and provide a broader context for the research. By following these comprehensive steps, the study generated valuable insights and knowledge that contributed to the sporting community and advanced the understanding of relevant topics within the field.

### 2.3. Instrument

In this study, the Coach-Athlete Relationship Questionnaire (CART-Q), developed by Yang and Jowett (2012), played a crucial role in assessing the athlete-coach relationship. This questionnaire allowed participants to rate their perceptions of the relationship on a Likert-type scale, ranging from 1 (Strongly Disagree) to 7 (Strongly Agree).

Translation of Instruments: The exacting translation of the research tool, the Coach-Athlete Relationship Questionnaire (CART-Q), created by Yang and Jowett (2012), and was a crucial component of this study. The translation procedure tried to maintain the semantic homogeneity of the questionnaire about the two different cultural and linguistic contexts of Spain and China.

The following steps were taken throughout the translation process:

(1) Forward Translation: A multilingual translator fluent in both languages performed the first translation of the CART-Q from its native language (English) into Spanish. This process aims to preserve cultural relevance and clarity while capturing the intended meanings of the questionnaire questions.

(2) Back Translation: A second bilingual translator unfamiliar with the original English version back translated the Spanish version of the CART-Q into English. This back translation ensured that the Spanish version’s semantic equivalent and substance stayed true to the original English text.

(3) Expert Review: To further improve the accuracy of the translations, linguists and scholars with extensive knowledge of the coach-athlete connection conducted a thorough analysis. This expert group evaluated the translated texts for clarity, cultural appropriateness, and semantic precision.

(4) Pilot Testing: A small group of people proficient in both languages (Spanish and Chinese) were used for the pilot test before the translated questionnaires were given to the participants (Diotaiuti et al., 2021). This pilot test aimed to identify and address any possible difficulties with understanding and cultural relevance.

Linguist Consultations: The linguists, academics, and cultural specialists engaged in the project had regular meetings and discussions throughout the translation process. Through these discussions, it was certain that the translated versions of the CART-Q appropriately reflected the subtleties of the constructions relating to the coach-athlete interaction in the Spanish and Chinese cultural settings (Yang, 2011).

### 2.4. Data analysis

Descriptive data is presented as mean ± standard deviation (SD). Also, frequencies and percentages were calculated. The reliability of the questionnaire in this survey was assessed using Cronbach’s alpha coefficient, yielding a value of 0.871, indicating good reliability. Thresholds were employed to determine the quality of reliability, with α ≥ 0.8 considered good, 0.8 > α ≥ 0.7 relatively good, 0.7 > α ≥ 0.6 acceptable, and α < 0.6 poor. *T*-tests, analysis of variance (ANOVA), and multivariate analysis of variance (MANOVA) were conducted to compare mean scores across different groups or conditions. Correlations between variables were examined quantitatively and qualitatively to understand their relationship comprehensively. Effect sizes were considered to assess the practical significance of findings. The significance level was set at *p* < 0.05, and the Statistical Package for the Social Sciences (SPSS) version 26 was utilized for analysis. These rigorous statistical procedures enhance the credibility and validity of the study’s findings, providing comprehensive insights into the coach-athlete relationship.

## 3. Results

The interpretation of the results is contextualized within the intricate interplay of Chinese and Spanish cultural frameworks. This study adopts a rigorous comparative approach, taking into account the cultural nuances inherent to China and Spain. These distinctive cultural frames can significantly influence the coach-athlete relationship, particularly in terms of closeness, commitment, and complementarity.

A thorough instrument validation procedure was rigorously carried out prior to data analysis to guarantee that the translated instruments were acceptable in both the Chinese and Spanish cultural settings. This validation process included several crucial procedures. A pilot testing phase was first conducted with several individuals from both cultures. This early evaluation might reveal any possible language and cultural understanding problems. Expert linguists and cultural specialists then worked together to conduct a detailed analysis of the translated instruments. Their priceless advice and input were very helpful in modifying the instruments so that they perfectly matched the distinctive language and cultural characteristics of both China and Spain. A thorough fit assessment confirms the validity and dependability of the instruments employed in this cross-cultural investigation, underscoring the dedication to thorough data collecting.

Significant differences were observed among age groups in the dimensions of closeness and complementarity (Table 1), as revealed by one-way analysis of variance (ANOVA). *Post hoc* LSD tests indicated that coaches and athletes in the age group over 21 achieved higher scores on all items than the ages of 14–16 and 17–20, suggesting an upward trend in scores as age increases. However, no significant differences were found among the age groups in the dimension of commitment, as indicated by a *p*-value of 0.124.

**TABLE 1:** One-way ANOVA analysis of variance for age group and each dimension.

Dimensions	Sum of squares	Mean square	Df	F	*P*	LSD
Closeness	17.334	8.667	2**	5.57	0.004*	3 > 2 > 1
Commitment	9.133	4.567	2**	2.09	0.124	3 > 2 > 1
Complementarity	24.405	12.203	2**	10.38	0.000	3 > 2 > 1

*1 = 14–16, **2 = 17–20, and **3 = over 21.

Examining the descriptive statistics in this part is vital because they provide a better understanding of the observed changes in the characteristics of the coach-athlete interaction between age groups. For each proximity and complementarity dimension, mean scores and standard deviations have been determined as part of the descriptive analysis. These data reveal subtle trends in the interaction between coaches and athletes. These descriptive measurements allow us to understand how the coach-athlete connection changes as athletes mature. In particular, mean scores provide a general picture of the main trends, while standard deviations show the degree of variation within each age group. This in-depth investigation complements the more general conclusions drawn from the ANOVA study by improving the understanding of how age affects the closeness and complementarity characteristics.

The results show that questions 3, 4, 5, 7, and 11 significantly differ between age groups (Table 2), while questions 1, 2, 6, 8, 9, and 10 do not. Additionally, looking at the means in Table 3, athletes from both countries over 21 scored higher on each question than those in the 14–16 and 17–20 age groups. This indicates that the scores on each question increase as the age group increases.

**TABLE 2:** One-way ANOVA analysis of age group and each question.

Item	df	F	*P*
3. I like my coach	2	11.32	0.000
4. I am at ease	2	11.42	0.000
5. I trust my coach	2	4.59	0.010
7. I am responsive to his/her efforts	2	10.36	0.000
11. I adopt a friendly stance	2	6.28	0.002

**TABLE 3:** Score differences of each question across age groups.

Item	Age Group	N	Mean	SD
3. I like my coach	14–16	77	4.17	1.824
	17–20	232	4.25	1.927
	over 21	462	4.89	1.87
4. I am at ease	14–16	77	3.94	1.765
	17–20	232	3.99	1.754
	over 21	462	4.58	1.707
5. I trust my coach	14–16	77	4.31	1.887
	17–20	232	4.28	1.759
	over 21	462	4.69	1.813
7. I am responsive to his/her efforts	14–16	77	4.78	1.804
	17–20	232	5.54	1.318
	over 21	462	5.52	1.31
11. I adopt a friendly stance	14–16	77	4.68	1.743
	17–20	232	5.19	1.479
	over 21	462	5.32	1.434

### 3.1. Multivariate analysis of variance (MANOVA)

The analysis of the data from Table 4 demonstrates a highly significant influence of country differences on the variables of “closeness,” “commitment,” and “complementarity.” These findings indicate notable disparities in the values of these variables between China and Spain, specifically regarding the closeness, commitment, and complementarity exhibited between coaches and athletes. Significant differences in the level of closeness across different age groups among coaches and athletes are evident. However, the impact of age grouping variables on “commitment” and “complementarity” is only significant at a 10% level. Moreover, the interaction between country and age grouping variables significantly affects “closeness,” suggesting that country differences substantially influence intimacy when accounting for different age groups. These findings are consistent with the descriptive analysis results. Gender grouping variables do not show a significant impact on “closeness,” “commitment,” and “complementarity” at a 5% level. However, at a 10% level, gender-grouping variables significantly influence the “complementarity” variable. Thus, gender is not a prominent factor influencing the relationship of “closeness,” “commitment,” and “complementarity” between coaches and athletes.

**TABLE 4:** Between-subjects effects test.

Source	Dependent variable	Type III Sum of squares	Degrees of freedom	Mean square	F	Significance
Effect	Closeness	497.71^a^	11	45.24	36.33	0.000
	Commitment	354.76^b^	11	32.25	26.34	0.000
	Complementarity	195.38^c^	11	17.76	18.40	0.000
Intercept	Closeness	7908.28	1	7908.28	6350.43	0.000
	Commitment	8923.70	1	8923.70	7290.50	0.000
	Complementarity	11339.51	1	11339.51	11751.81	0.000
Country	Closeness	129.76	1	129.76	104.20	0.000
	Commitment	119.08	1	119.08	97.29	0.000
	Complementarity	51.68	1	51.68	53.56	0.000
Age group	Closeness	23.37	2	11.68	9.38	0.000
	Commitment	6.25	2	3.12	2.55	0.078
	Complementarity	5.31	2	2.65	2.75	0.064
Gender	Closeness	1.09	1	1.09	0.88	0.348
	Commitment	0.54	1	0.54	0.44	0.503
	Complementarity	3.56	1	3.56	3.69	0.055
Country × age group	Closeness	33.60	2	16.80	13.49	0.000
	Commitment	15.29	2	7.64	6.24	0.002
	Complementarity	12.54	2	6.27	6.50	0.002
Country × gender	Closeness	2.19	1	2.19	1.76	0.184
	Commitment	3.90	1	3.90	3.19	0.074
	Complementarity	6.99	1	6.99	7.24	0.007
Age group × gender	Closeness	0.60	2	0.30	0.24	0.786
	Commitment	2.65	2	1.32	1.08	0.338
	Complementarity	3.88	2	1.94	2.01	0.134
Country × age group × gender	Closeness	7.10	2	3.55	2.85	0.058
	Commitment	11.74	2	5.87	4.79	0.009
	Complementarity	10.79	2	5.39	5.59	0.004
Error	Closeness	935.23	751	1.24		
	Commitment	919.23	751	1.22		
	Complementarity	724.65	751	0.96		
Total	Closeness	17154.87	763			
	Commitment	18521.66	763			
	Complementarity	22483.81	763			
Corrected total	Closeness	1432.94	762			
	Commitment	1274.00	762			
	Complementarity	920.03	762			

a. R-squared = 0.347 (Adjusted R-squared = 0.33).

b. R squared = 0.278 (Adjusted R-squared = 0.26).

c. R squared = 0.212 (Adjusted R-squared = 0.20).

It is crucial to note that even though the mediation analysis and structural equation model are mentioned in the abstract, this particular analysis was not part of the current investigation. Instead, the findings from the ANOVA and MANOVA analyses are the main emphasis of this study. It was decided not to undertake the mediation analysis for practical reasons, such as practical restrictions and sample size. Although the mediation study was abandoned, the impactful results from the ANOVA and MANOVA studies will be discussed in the following sections, providing important insights into the dynamics of the coach-athlete interaction within the examined cultural frameworks.

### 3.2. Multiple comparison

The following multiple comparison Table 5 provides the results of the comparisons based on the age group variable. From the table, the following are the observations:

**TABLE 5:** Multiple comparison.

Dependent variable	(I) age group	(J) age group	Mean difference (I-J)	Standard error	Significance	95% Confidence interval	
						Lower bound	Upper bound
**LSD**							
Closeness	14–16	17–19	−0.15	0.14	0.30	−0.44	0.13
		Over21	−0.60*	0.13	0.00	−0.87	−0.32
	17–19	14–16	0.15	0.14	0.30	−0.13	0.44
		Over21	−0.44*	0.09	0.00	−0.62	−0.26
	Over21	14–16	0.60*	0.13	0.00	0.32	0.87
		17–19	0.44*	0.09	0.00	0.26	0.62
Commitment	14–16	17–19	−0.18	0.14	0.20	−0.47	0.10
		Over21	−0.35*	0.13	0.01	−0.62	−0.08
	17–19	14–16	0.18	0.14	0.20	−0.10	0.47
		Over21	−0.16	0.08	0.06	−0.34	0.01
	Over21	14–16	0.35*	0.13	0.01	0.08	0.62
		17–19	0.16	0.08	0.06	−0.01	0.34
Complementarity	14–16	17–19	−0.23	0.13	0.07	−0.48	0.02
		Over21	−0.34*	0.12	0.00	−0.58	−0.10
	17–19	14–16	0.23	0.13	0.07	−0.02	0.48
		Over21	−0.10	0.07	0.17	−0.26	0.04
	Over21	14–16	0.34*	0.12	0.00	0.10	0.58
		17–19	0.10	0.07	0.17	−0.04	0.26

The error term is the Mean Square (Error) = 0.96 based on the observed mean values.

*The significance level of the mean difference is.05.

Firstly, regarding the impact on “Closeness,” there is a significant difference in the effects of the age group “14–16” compared to the age group “21 and above” on the closeness between coaches and athletes. Additionally, there is a significant difference in the impact of the age group “17–19” compared to the age group “21 and above” on closeness. However, the distinction in the effects of the age group “14–16” compared to “17–19” on closeness between coaches and athletes is not very pronounced. Secondly, concerning the impact on “Commitment,” there is a significant difference in the effects of the age group “14–16” compared to the age group “21 and above” on the commitment between coaches and athletes. Lastly, regarding the impact on “Complementarity,” there is a significant difference in the effects of the age group “14–16” compared to the age group “21 and above” on the complementarity between coaches and athletes.

## 4. Discussion

Enhancing athlete contentment and general wellbeing requires a thorough understanding of the dynamics of the coach-athlete interaction. In federated basketball, an athlete’s success and performance depend on their connection with their coach. The Coach-Athlete Relationship Questionnaire (CART-Q) was used in this research to assess the emotional resemblance, dedication, and complementary nature of coaches and athletes in China and Spain.

In reviewing the main outcomes of this research, it is important to note that, especially in the context of federated basketball, improving player happiness and general wellbeing requires an awareness of the dynamics of the coach-athlete relationship. The Coach-Athlete Relationship Questionnaire (CART-Q) was used in this research to assess the emotional compatibility, dedication, and complementarity between coaches and athletes in China and Spain.

This research aims to further shed light on the influence of cultural and contextual variables on coaching methods and player-coach interactions, building on the insightful findings of Freire et al. (2023), who examined the coach-athlete connection in two different nations. The results are comparable with Zhang et al. (2020), who examined the data and found that Spanish athletes regularly outperformed athletes from an unidentified nation in all three aspects of the coach-athlete interaction. This finding shows that interactions between coaches and athletes in Spain are more solid and encouraging than those in the unnamed nation.

The coaching methods used by Spanish coaches, a phenomenon well-documented in earlier studies, provide one tenable explanation for these disparities. A friendly and supportive coaching atmosphere is fostered by Spanish coaches’ player-centred and collaborative coaching methods. Building mutual respect, open communication, and trust between coaches and athletes is emphasized in this method (MacLennan, 2017). In Spain, coaches routinely include players in decision-making processes and value their feedback on training plans and game strategy. These activities give athletes ownership and accountability, strengthening their dedication to their growth and the team’s success.

Additionally, the larger Spanish culture significantly shapes the connection between the coach and the player. Spanish culture places a strong priority on personal connections, and this concept naturally permeates sports. Spanish coaches are more likely to consider players as different persons with specific needs and goals. As a result, they often provide more specialized coaching and assistance. On the other hand, the study’s undisclosed nation could have distinct cultural standards and coaching culture that affect how coaches and players interact (Flaherty, 2022). Consequently, interactions may become less encouraging and cooperative and adopt a more hierarchical or authoritarian coaching style with less space for athlete input and specialized care.

These results provide insightful information regarding the coaching environment in China, particularly in light of the lower scores shown among Chinese athletes across all three aspects of the coach-athlete relationship. These differences, in conjunction with the findings of Giulianotti (2015), highlight the significant influence that cultural differences have on coaching methods and relationships between athletes and coaches. Cultural variations profoundly affect how players communicate, interact with coaches, and see authoritative figures.

People often value collective peace and conformity in collectivist countries like China, which might affect the dynamics of coach-athlete interaction. Without expressing, their feelings aloud, athletes may be more likely to comply with instructions, and collaboration with coaches may be shown by compliance and adherence to set rules (Jowett, 2017). While deference to authority is important, coaches must also provide a loving and encouraging atmosphere where players feel free to express themselves and offer suggestions. The coaching experience and athlete performance may be improved through better communication and mutual understanding between coaches and athletes.

Coaches, sports organizations, and officials in China must be aware of these cultural impacts. Putting techniques into practice that improve the coach-athlete connection and cross-cultural divides is crucial. Chinese coaches may foster a more collaborative and player-focused atmosphere by highlighting the advantages of open communication, shared trust, and personalized attention. Coaches may allow players to share their thoughts and ideas with the training and competition processes to encourage ownership and engagement.

The study’s research on how age affects the connection between coaches and athletes sheds important light on how players’ viewpoints and interactions with coaches change as they age and acquire experience. According to research by Kalén et al. (2020), coach-athlete interactions tend to become tighter and more cooperative as athletes develop and improve. Higher ratings for complementarity and intimacy among adult athletes demonstrate this (Wachsmuth et al., 2017). Due to variables including enhanced trust, a better grasp of one another’s viewpoints, and a stronger alignment of aims, older athletes often demonstrate improved attitudes toward their instructors (Gorgulu, 2019). A more robust and encouraging coach-athlete connection may arise when players become more receptive to collaborative efforts with their coaches as they acquire experience and confidence in their talents.

The study found that commitment ratings did not significantly change among age groups, which is interesting. This implies that players of all ages continue to show the same commitment and devotion to their coaches, highlighting the lasting relationship between athletes and their instructors regardless of age or degree of expertise. Another important result is the lack of significant gender variations in the parameters of coach-athlete relationships in China and Spain (Hong and Li, 2023). This suggests equal coaching methods since it shows that male and female athletes have similar closeness, commitment, and complementarity with their coaches (Fransen et al., 2017). This gender equality is heartening because it shows supportive coaching settings that appreciate and assist athletes of both sexes (Maguire, 2010).

The findings of this inquiry have important ramifications for the basketball associations in China and Spain. Sports organizations may better meet the requirements of players and provide a healthy coaching environment by taking into account the variations in the coach-athlete interaction between the two nations (Sugden and Tomlinson, 2018). Leveraging the positive aspects of Spain’s player-focused and cooperative coaching methods may be a springboard for further initiatives to strengthen the encouraging coach-athlete connection, eventually improving athlete happiness and performance.

Despite these significant findings, it is essential to acknowledge the limitations of this study. The stratification of the participant sample is one restriction. Although the research focused on the coach-athlete connection in the federated basketball community, a more thorough sample segmentation would provide more insightful results. Comprehension of the interaction between coaches and athletes may be improved by considering several subcategories of athletes regarding their ages, competition levels, kinds of sports, and training loads.

Particularly, the element of training load has the potential to significantly increase heterogeneity, perhaps resulting in varying views across athletes, even within the same sport. Variations in training loads may have a big influence on relationships between athletes and coaches, as Lupo et al. (2017) show in their study on female basketball players. This emphasizes the need for more studies to thoroughly examine how training load affects coach-athlete interactions.

The coach-athlete connection aspects’ lower ratings in China suggest areas for improvement. These results should not be seen as limitations but as catalysts for more research into coaching approaches and cultural factors. These assessments may shed light on the causes of discrepancies and provide information on what needs to be done to address them. The theory and practices of coaching in China are a topic that needs more investigation. Understanding how coaches approach motivating, enhancing, and developing athletes may help identify areas where beneficial changes can be made (Tan and Bairner, 2011). Coaches may consider using strategies that put players’ needs and viewpoints first, encouraging teamwork, honest communication, and developing trust and respect between coach and athlete.

Understanding how the coach-athlete connection affects performance, motivation, and general wellbeing. Within federated basketball and other sporting contexts, coaches have a crucial role in determining player development and pleasure. Coaches can help players feel valued, understood, and inspired to reach their greatest potential by fostering a pleasant and encouraging atmosphere. A systematic and trustworthy tool for evaluating the closeness, commitment, and complementarity in the coach-athlete connection is the Coach-Athlete Connection Questionnaire (CART-Q). The all-encompassing approach has made it easier to gather insightful information on the psychological, cognitive, and behavioral components of interactions between athletes and coaches, leading to a full knowledge of this phenomenon.

The observed differences in coach-athlete relationships between China and Spain highlight the importance of cultural and environmental elements in coaching tactics. Coaches may successfully connect with athletes from various cultural backgrounds by being aware of cultural nuances and adapting their coaching strategies appropriately. The greater performance of Spanish athletes demonstrates the value of coaching strategies that emphasize each player individually and promote teamwork. The relatively lower results among Chinese athletes provide a chance for growth and improvement in China’s coaching industry. These results must be catalysts for additional in-depth research into coaching approaches and cultural factors rather than limitations. These assessments may shed light on the causes of discrepancies and provide information on what needs to be done to address them.

For coaches, sports organizations, and legislators, comprehending the coach-athlete connection’s effects is crucial. Accepting cultural diversity and using coaching techniques that are appropriate for the situation may improve an athlete’s performance. Coaches should modify their teaching strategies as players go through their sports careers to match each athlete’s specific needs and goals while establishing a supportive and welcoming coaching atmosphere (López de Subijana et al., 2021).

This research helps comprehend the coach-athlete connection in federated basketball in China and Spain’s cultural and environmental differences. Cultural and coaching methods influence athlete-coach relationships, as seen by the inequalities. Sports organizations may improve athlete happiness and performance by recognizing these variations and using culturally sensitive coaching methods. Coaches, sports organizations, and politicians must understand the coach-athlete interaction. Cultural diversity and context-specific coaching improve athlete happiness, motivation, and performance. In positive and inclusive coaching settings, coaches should adapt to players’ specific needs and expectations at various phases of their athletic experiences.

## 5. Conclusion

In this particular investigation, the Coach-Athlete Relationship Questionnaire (CART-Q) was utilized to examine the dynamics of interactions between players and coaches and the relationships among players from China and Spain in the context of basketball. The findings revealed significant differences between the two nations, indicating that cultural and contextual factors are pivotal in shaping the coach-athlete relationship dynamics.

Notably, athletes from Spain exhibited higher scores, indicating stronger and more supportive associations with their coaches. In comparison, their counterparts from China obtained lower scores, signaling the potential for enhancing development opportunities. Additionally, the research discovered that older athletes tended to experience higher levels of closeness and complementarity with their coaches, which suggests a positive evolution in players’ attitudes toward their coaches. Noteworthy, too, is that the unwavering dedication across various age groups implies that athletes of all ages are equally committed to their coaches.

An intriguing discovery from the study was the absence of significant gender differences in the coach-athlete relationship dimensions in both China and Spain. This suggests that male and female athletes receive equal coaching support, regardless of gender. The findings emphasize the importance of cultivating positive and supportive coach-athlete relationships while considering cultural and age-related factors. The implications of this finding underscore the importance of developing positive and supportive coach-athlete relationships while considering cultural and age-related aspects.

Addressing these factors and promoting equitable coaching practices can profoundly affect the athlete’s experience and development. To more effectively and promote equitable coaching practices, there is a clear need for a thorough analysis of the athlete sample. This necessitates a comprehensive stratification of participants, taking into account various subcategories of athletes based on factors such as age, competition level, type of sport, and training load. This nuanced approach has the potential to reveal disparities in the perception of training load, even among individual athletes during different training sessions, a phenomenon previously underscored in research by Lupo et al. (2017) and further substantiated by their 2020 investigation into female basketball players.

The insights gleaned from this study hold significant value as practical guidance for sports organizations seeking to refine their coaching strategies and cultivate an environment that is both inclusive and supportive for athletes. Looking ahead, future research in the realm of federated basketball and other sporting contexts can build upon these findings. This research should explore additional variables that influence the coach-athlete relationship while also examining coaching practices across diverse sporting cultures. In this endeavor, drawing inspiration from the body of work by Lupo et al. (2020) is highly relevant.

Practical interventions based on these research findings can be implemented to enhance coach-athlete relationships within sports organizations. Prioritizing the development of cultivating healthy and effective connections between coaches and athletes is pivotal, as it can culminate in a positive and enriching athletic experience. Ultimately, this has a ripple effect, benefiting not only the athletes but also coaches and the sports institutions they are affiliated with.

## Data availability statement

The raw data supporting the conclusions of this article will be made available by the authors, without undue reservation.

## Ethics statement

The studies involving humans were approved by the Ethics Committee of the Polytechnic University of Madrid. The studies were conducted in accordance with the local legislation and institutional requirements. Written informed consent for participation in this study was provided by the participants’ legal guardians/next of kin.

## Author contributions

JW: Data curation, Investigation, Writing—original draft. CC-M: Supervision, Writing—review and editing. JO-B: Methodology, Supervision, Writing—review and editing.
